# ”Hope Dies Last”: Older FSU Immigrants Displaced from the Gaza Envelope During the Israel Hamas War

**DOI:** 10.1093/geroni/igaf122.3255

**Published:** 2025-12-31

**Authors:** Ksenya Shulyaev, Tova Band-Winterstein, Pnina Dolberg, Gabriela Spector-Mersel

**Affiliations:** University of Haifa, Haifa, HaZafon, Israel; University of Haifa, Haifa, HaZafon, Israel; The Ruppin Academic Center, Kfar Monash, HaMerkaz, Israel; Sapir College, Sderot, HaDarom, Israel

## Abstract

Introduction The October 7th Hamas attack and the subsequent war led to the immediate evacuation of tens of thousands of residents from the Gaza Envelope. Among them were older immigrants from the Former Soviet Union (FSU) who had lived in a Continuous Traumatic Situation (CTS) for years. This study explores the unique experiences of these displaced older adults, examining the intersections of aging, migration, and prolonged exposure to conflict. Methods This qualitative-narrative study is based on in-depth interviews with ten FSU immigrants (both men and women), aged 75+, who lived in the Gaza Envelope for at least a decade and were displaced following the October 7th attack. The study employed semi-structured interviews to examine their lived experiences of forced displacement, past immigration, and long-term exposure to trauma. Results Four key themes emerged: (1) From trauma to accumulated trauma—a lifetime of exposure to war-related events, (2) “You’re not a child anymore. You’re an adult—pull yourself together”—self-discipline and emotional regulation as coping mechanisms, (3) Life in limbo between alienation and belonging—navigating identity as older migrants in a conflict zone, and (4) Social networks as resilience anchors—the vital role of community and structured support in coping with displacement. Conclusions The study highlights the complex interplay between past and present traumas in the lives of older FSU immigrants living near Gaza. Findings underscore the need for targeted interventions that enhance resilience, foster a sense of continuity, and provide tailored support to older displaced immigrants facing prolonged adversity.

